# *In vitro* model of postoncosphere development, and *in vivo* infection abilities of *Taenia solium* and *Taenia saginata*

**DOI:** 10.1371/journal.pntd.0007261

**Published:** 2019-03-14

**Authors:** Sandra Palma, Nancy Chile, Rogger P. Carmen-Orozco, Grace Trompeter, Kayla Fishbeck, Virginia Cooper, Laura Rapoport, Edson G. Bernal-Teran, Beth J. Condori, Robert H. Gilman, Manuela R. Verastegui, for the Cysticercosis Working Group in Peru

**Affiliations:** 1 Infectious Diseases Research Laboratory, Department of Cellular and Molecular Sciences, Faculty of Sciences and Philosophy, Universidad Peruana Cayetano Heredia, Lima, Peru; 2 Department of International Health, Bloomberg School of Hygiene and Public Health, Johns Hopkins University, Baltimore, Maryland, United States of America; 3 Department of Global Health and Population, Harvard T.H. Chan School of Public Health, Harvard University, Boston, United States of America; Queen's University Belfast, UNITED KINGDOM

## Abstract

*Taenia solium* is known to cause human cysticercosis while *T*. *saginata* does not. Comparative *in vitro* and *in vivo* studies on the oncosphere and the postoncospheral (PO) forms of *T*. *solium* and *T*. *saginata* may help to elucidate why cysticercosis can occur from one and not the other. The aim of this study was to use *in vitro* culture assays and *in vivo* models to study the differences in the development of the *T*. *solium* and *T*. *saginata* oncosphere. Furthermore, this study aimed to evaluate the expression of cytokines and metalloproteinases (MMPs) in human peripheral blood mononuclear cells (PBMCs), which were stimulated by these oncospheres and PO antigens. *T*. *solium* and *T*. *saginata* activated oncospheres (AO) were cultured in INT-407 and HCT-8 intestinal cells for 180 days. The *T*. *solium* began to die while the *T*. *saginata* grew for 180 days and developed to cysticerci in INT-407 cells. Rats were inoculated intracranially with AO and PO forms of either *T*. *saginata* or *T*. *solium*. Rats infected with *T*. *solium* AO and PO forms developed neurocysticercosis (NCC), while those infected with the *T*. *saginata* did not. Human PMBCs were stimulated with antigens of AO and PO forms of both species, and the production of cytokines and metalloproteinases (MMPs) was measured. The *T*. *solium* AO antigen stimulated a higher production of IL-4, IL-5, IL-13, IFN-γ, and IL-2 cytokines compared to *T*. *saginata* AO. In the PO form, the *T*. *saginata* PO antigen increased the production of IL-4, IL-5, IL-13, IFN-γ, IL-1β, IL-6, IL-10, TNF-α and IL-12 cytokines compared to *T*. *solium*, suggesting that this global immune response stimulated by different forms could permit survival or destruction of the parasite depending of their life-cycle stage. Regarding MMPs, *T*. *solium* AO antigen stimulated a higher production of MMP-9 compared to *T*. *saginata* AO antigen, which may be responsible for altering the permeability of intestinal cells and facilitating breakdown of the blood-brain barrier during the process of invasion of host tissue.

## Author summary

*Taenia solium* and *Taenia saginata* are two parasites that cause the tissue infection cysticercosis in their intermediate hosts, pigs and cows, respectively. One major difference between them is that *T*. *solium* can also cause neurocysticercosis in the human brain, while *T*. *saginata* cannot. Neurocysticercosis is thought to be the major cause of adult-onset seizures in developing countries. It is not well understood why only *T*. *solium* can survive in human tissue; however, the host inflammatory response likely plays an important role. The authors found that human immune cells stimulated with *T*. *solium* in the early stages of the parasite life cycle produced a more robust cytokine response than *T*. *saginata*. However, in the mature stage, which occurs once *T*. *solium* reaches the brain, *T*. *solium* antigens stimulated a lower inflammatory response compared to *T*. *saginata*, suggesting the parasite is able to manipulate the host immune response in some way to evade destruction. These findings may support the differences in growth observed by the authors when rat brains were inoculated with either parasite species. This study provides new insights into the different ways *T*. *solium* and *T*. *saginata* activate the immune response to survive and develop within the host.

## Introduction

*Taenia solium* and *T*. *saginata* are two taeniid cestodes that cause the diseases taeniasis and cysticercosis [1]. These are zoonotic diseases, and swine and bovine act as intermediate hosts, causing porcine and bovine cysticercosis, respectively. Humans act as the definitive hosts in both *T*. *solium* and *T*. *saginata* infection leading to taeniasis. In the case of *T*. *solium*, humans can also act as accidental intermediate hosts causing human cysticercosis [2]. However, only *T*. *solium* causes human cysticercosis, while *T*. *saginata* does not [3].

When cysticercosis involves the central nervous system in humans, it is called neurocysticercosis (NCC). NCC is common throughout Latin America, sub-Saharan Africa, most of Asia, and parts of Oceania. Human NCC is believed to be the leading cause of acquired epilepsy worldwide [4,5].

The eggs of *T*. *solium* and *T*. *saginata* contain a six-hooked larva called the oncosphere [6]. When the eggs hatch, this oncosphere is released into the intestine. Intestinal fluid dissolves the oncospheral membrane, releasing and activating the oncosphere. The *T*. *solium* activated oncosphere can then penetrate the intestinal wall. Once in the tissue, usually in the muscle or the central nervous system, the oncosphere can transform into a postoncospheral form, and completely develop into cysticerci—a larval stage that consists of a fluid-filled sac containing an invaginated scolex. When this happens, the parasite produces a variety of molecules, which modulate the host immune response in order to evade parasite destruction [7].

The postoncospheral (PO) form is an intermediate stage between an oncosphere and a fully developed cysticercus in tissue [8]. The PO form of *T*. *solium* and *T*. *saginata* can be obtained *in vitro* by co-culture of oncospheres with a monolayer of mammalian feeder cells [9,10]. *T*. *solium* oncosphere and *in-vitro* generated PO forms can develop into cysticercus in rats causing NCC [9,11]. However, little is known regarding the *in vitro* development of the oncosphere to PO form of *T*. *solium* and *T*. *saginata*, specifically the immunological events that occur at the host/parasite interface. Also, it is not known if the development of the *T*. *saginata* oncosphere to PO form could cause NCC in the rat model as *T*. *solium* does. *In vitro* and *in vivo* models could lead to a better understanding of host-parasite relationships.

Host immune cells such as macrophages, lymphocytes, and polymorphonuclear leukocytes can produce cytokines and metalloproteinases (MMPs) in order to prevent the development of the parasite [12,13]. Because of this, the parasite has developed mechanisms to evade or modulate the host immune response.

Comparative studies on the oncosphere and the PO form of *T*. *solium* and *T*. *saginata* are limited. This study focused on the *in vitro* development of the oncosphere to the PO form for *T*. *solium* and *T*. *saginata*. These *in vitro*-developed larvae were then tested for infectivity in rats. Later, we obtained antigens from the *T*. *solium* and *T*. *saginata* oncosphere and PO forms to stimulate the production of cytokines and MMPs in healthy human peripheral blood mononuclear cells (PBMCs).

## Materials and methods

### Cell culture preparation

HCT-8 and INT-407 cells, obtained from the American Tissue Culture Collection (ATCC, Manassas, VA), were used to obtain *T*. *solium* and *T*. *saginata* PO forms. Cells were incubated at 37°C in 5% CO_2_ and grown in a specific medium as recommended by ATCC (EMEM media for INT-407, and RPMI for HCT-8; all medium was supplemented with 10% fetal bovine serum). The medium was changed every two days. Once cell confluency was obtained, cells were harvested using trypsin-EDTA (Sigma Chemical Co). Cells were placed into 24-well plates (1x10^5^ cells per well) for maturation assay. The assays described below were performed when cells formed a monolayer.

### *Taenia solium* and *T*. *saginata* oncosphere preparation

Tapeworms were collected after medical treatment of newly diagnosed patients, as described by Jeri *et al* [14]. Hatching of eggs and oncosphere activation were performed, as described by Verastegui *et al* [15]. The eggs were obtained from gravid proglottids of adult tapeworms by gentle homogenization in a 2.5% potassium dichromate solution (Sigma, St. Louis, Missouri). Eggs were then washed three times in distilled water with centrifugation steps to collect the eggs between washes in 2500g for 5 minutes. The eggs were hatched, and the oncospheres were released using a solution of 0.75% sodium hypochlorite in water for 10 minutes (Mallinckrodt Baker, Inc, Phillipsburg, NJ). Oncospheres were then washed three times in RPMI medium (Sigma, St. Louis, Missouri), and activated by incubation at 37°C for 45 minutes (in the case of *T*. *solium)* or 90 minutes (in the case of *T*. *saginata)* with artificial intestinal fluid (1 g pancreatin (Sigma Chemical Co., St. Louis, MO), 200 mg Na_2_CO_3_, and 1 ml of fresh porcine bile (for *T*. *solium*), or 1 ml of fresh bovine bile (for *T*. *saginata*), with enough RPMI 1640 medium (pH 8.04) to make 100mL). After activation, the oncospheres were washed three times with RPMI medium and counted using a Neubauer chamber.

### *In vitro* maturation assay

Parallel *in vitro* maturation assays with INT-407 and HCT-8 monolayer cells, using *T*. *solium* and *T*. *saginata* activated oncospheres, were conducted in order to compare the morphological characteristics during development of each species using the methodology reported by Chile *et al* [9]. Ten thousand activated oncospheres were cultured in confluent INT-407 and HCT-8 monolayer cells for two weeks. During that time, the medium was changed every three days. At day 15 of culture, the postoncospheral forms were collected and rinsed twice with fresh medium, then transferred to another well containing a confluent of monolayer cells. This process was repeated every three days for up to six months to allow the postoncospheral forms to continue to develop. Cultures were inspected daily using an inverted microscope (Leitz labovert FS). Parasites were collected at 15, 30, 60, 120, and 180 days of incubation.

### Infection of rats with *T*. *solium* and *T*. *saginata* activated oncosphere (AO) and PO forms

To determine if *T*. *saginata* AO and PO forms can develop into viable cysts *in vivo*, 15-day old Holtzman rats, purchased from Universidad Peruana Cayetano Heredia, Lima, Peru, were infected intracranially (in the bregma) with oncospheres and 15-day old PO forms from either species following the methodology reported by Verastegui et al., 2015 [11]. Rats were anaesthetized with ketamine (100 mg/kg body weight) and xylazine (5 mg/kg body weight) before infection.

Six rats were inoculated with 180 *T*. *solium* AO in 100 μL of saline solution; seven rats were inoculated with 180 *T*. *saginata* AO in 100 μL of saline solution. The negative control was 2 rats inoculated with saline solution.

Eight rats were inoculated with ten *T*. *solium* 15-day PO forms in 100 μL of saline solution, eight rats were inoculated with ten *T*. *saginata* 15-day PO forms in 100 μL of saline solution, and five rats were inoculated with 100 μL of saline solution as a control. A 24-gauge syringe needle was used.

After four months, necropsy was performed. Rats were anaesthetized with ketamine (100 mg/kg body weight) and xylazine (5 mg/kg body weight). Anaesthetized rats were perfused with 200 ml of PBS and then with 100 ml of 4% paraformaldehyde in PBS. Brains were carefully removed, post-fixed for 24 hours at 4°C with 4% paraformaldehyde in PBS, and stored in 70% ethanol. Brains were observed macroscopically to identify extraparenchymal cysticerci. Five millimeter coronal brain sections were cut until the intraparenchymal cysticerci were observed.

### Antigen preparation

Antigens were obtained from AO and 30 day-PO forms of both parasites. AO and PO were obtained as described above. Parasites were rinsed three times with PBS buffer, sonicated, and centrifuged at 10,000g for 15 min at 4°C. The supernatant (total soluble antigens) was separated, and proteins were quantified using Bradford Protein Assay (Bio-Rad) and stored at -70°C until ready for use.

### *In vitro* stimulation of peripheral blood mononuclear cells (PBMCs)

Healthy volunteers (n = 13) with negative serology for NCC were invited to participate in this study. After the volunteers signed an informed consent form, 10 mL of venous blood was collected from each non-infected donor. PBMCs were collected from 10 ml EDTA blood. The blood samples were centrifuged at 400g on a Ficoll-Hypaque gradient (Ficoll–Paque ^TM^ PLUS, GE Healthcare) for 10 minutes at room temperature for the separation of mononuclear cells. Cells were again suspended in RPMI medium plus 5% of inactivated human serum. Cell viability was evaluated with trypan blue and counted in a Neubauer chamber. Each well contained 2x10^5^ group of cells was cultured in a 96-well plate at 37°C with 5% CO_2_ for 48 hours in an RPMI medium containing 5% of inactivated human serum. Cells were stimulated with 5 μg/ml of phytohemaglutinin (PHA) as the positive control, 20 μg/ml of AO antigens (both species, separately), and 20 μg/ml of antigens from PO forms at 30 days of maturation (both species, separately). At the end of the incubation period, PBMCs were harvested and centrifuged for 10 min at 400g, and supernatants were collected and stored at −70°C until tested for cytokine and metalloproteinase content by multiplex analysis.

### Cytokine and Metalloproteinases (MPP) assays

MILLIPLEX MAP kit High Sensitivity Human Cytokine Magnetic Bead Panel (Millipore) was used to measure cytokines (IFN-γ, IL-1β, IL-2, IL-4, IL-5, IL-6, IL-10, IL-12p70, IL-13 and TNF-α), and Fluorokine MAP (R&D system, Minneapolis USA) was used to measure MMP (MMP-2 and MMP-9) in the supernatant of stimulated PBMCs following the manufacturer’s instructions. The cytokines and MMP were detected by the Bio-plex 200 system (Bio-Rad Laboratories, Hercules, CA) using Luminex xMAP technology.

### Ethical statement

Experiments were done according to the Guide for care and Use of Laboratory Animals of the National Institutes of Health. The protocol was approved by the Committee on the Ethics Animals of the Universidad Peruana Cayetano Heredia, Lima, Peru (Permit Numbers: 61242).

### Data analysis

For cytokines and MMP assays, the median fluorescent intensities of the beads with the cytokines or MMP now bound were converted to concentrations (pg/ml), using a five-parameter logistic model using the Bio-Plex Manager™ 6.0 software (Bio-Rad Laboratories, Hercules, CA). Each MMP´s or cytokine’s concentration was normalized by subtracting the data of medium alone (control). The Mann Whitney test was used to compare two unpaired groups, and ANOVA followed by Tukey’s post-test was used for three or more group using the Prism V 6.0 statistical program (GraphPad). A P-value of <0.05 was considered statistically significant.

## Results

### *In* vitro development of *T*. *saginata* and *T*. *solium* from oncosphere

*T*. *saginata* and *T*. *solium* developed from the oncosphere to the PO form in both cell lines, INT-407 and HCT-8 cells (Table 1). In HCT-8 cells, both species of PO forms developed until 60 days of culture, and then began to die. However, after 60 days in INT-407 cells, the *T*. *saginata* PO forms continued to develop to cysticerci, while the *T*. *solium* PO forms began to die. At 180 days of culture 0.25% of *T*. *saginata* oncospheres developed to cysticerci.

**Table 1:** Oncospheres of *T*. *solium* and *T*. *saginata* cultured in HCT-8 and INT-407 cells during 180 days.

Day of culture	HCT-8 cells		INT-407 cells	
	*T*. *solium*	*T*. *saginata*	*T*. *solium*	*T*. *saginata*
15	PO form	PO form	PO form	PO form
30	PO form	PO form	PO form	PO form
60	PO form	PO form	PO form	PO form with structures like scolex
120	--	--	--	Cyst with scolex evaginated
180	--	--	--	Cyst with scolex evaginated

-- = Died; PO = Postoncosphere

**Fig 1:**
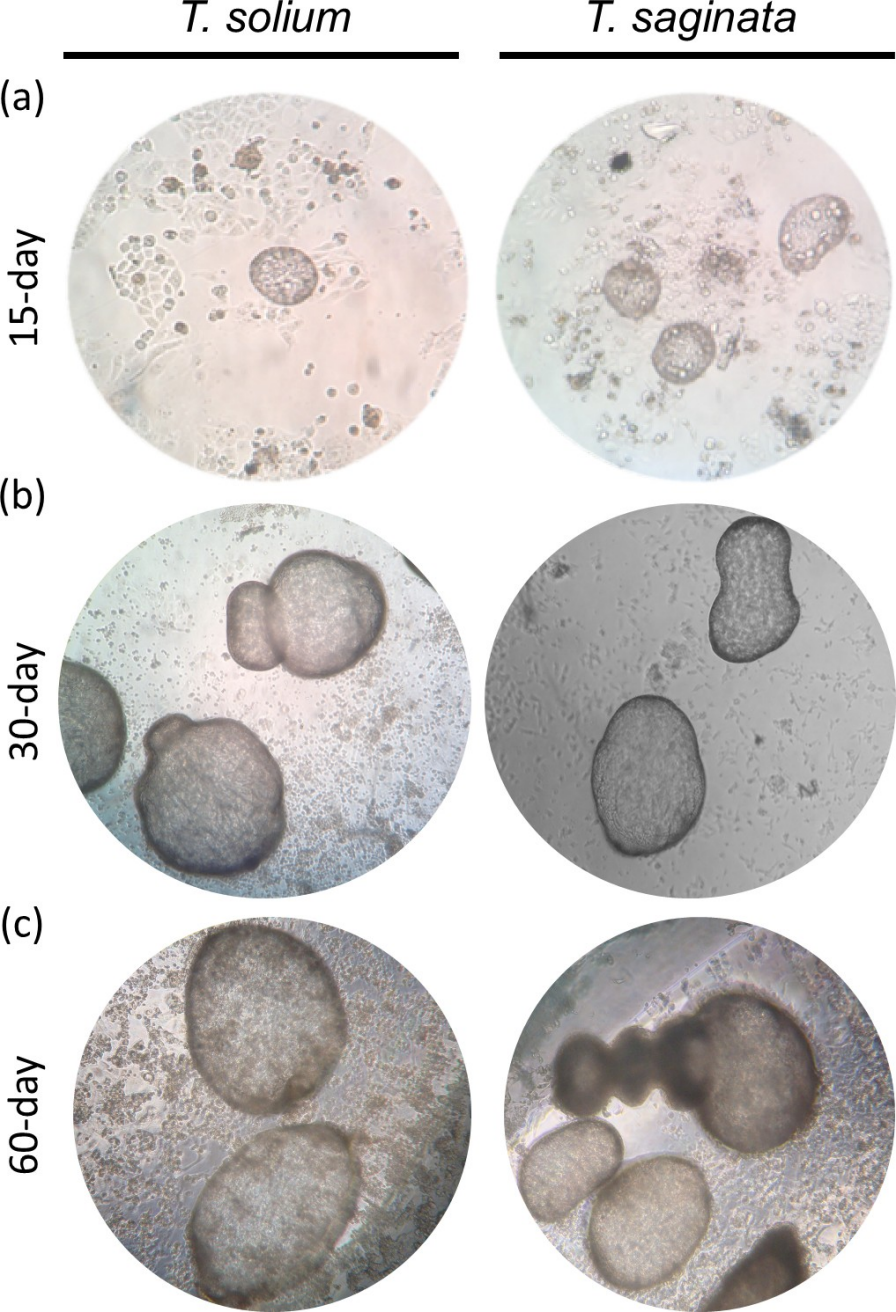
Light microscopy of postoncospheral form of *T*. *solium* and *T*. *saginata* displaying parasite morphology at different times of development in INT-407 culture. *T*. *solium* and *T*. *saginata* at 15-day PO form (a) 30-day PO form (b) and at 60-day PO form (c) Magnification 100x.

The morphological characteristics of the *T*. *saginata* and *T*. *solium* PO forms obtained from culture in INT-407 monolayer cells were compared. At 15 days of incubation, both species were morphologically similar (Fig 1A), ranging between 60 to 250 μm in size. Both had an oval form without hooks and shared characteristic movement. For both species at 30 days of incubation, PO forms increased in size (between 200 to 1000 μm), maintained their oval form, and formed a protuberance at one end of the body (Fig 1B).

At day 60 of culture, the *T*. *solium* PO form had a spherical shape, similar to a cyst without a scolex, and cells accumulated in the protuberance of one end of the body (Fig 1C). The *T*. *saginata* PO form had similar characteristics but also showed a neck-like prolongation at one end of the body (Fig 1C), reaching up to 3000 μm. Additionally, at day 60, *T*. *solium* PO forms began to die while *T*. *saginata* PO forms continued growing until 180 days of culture.

**Fig 2:**
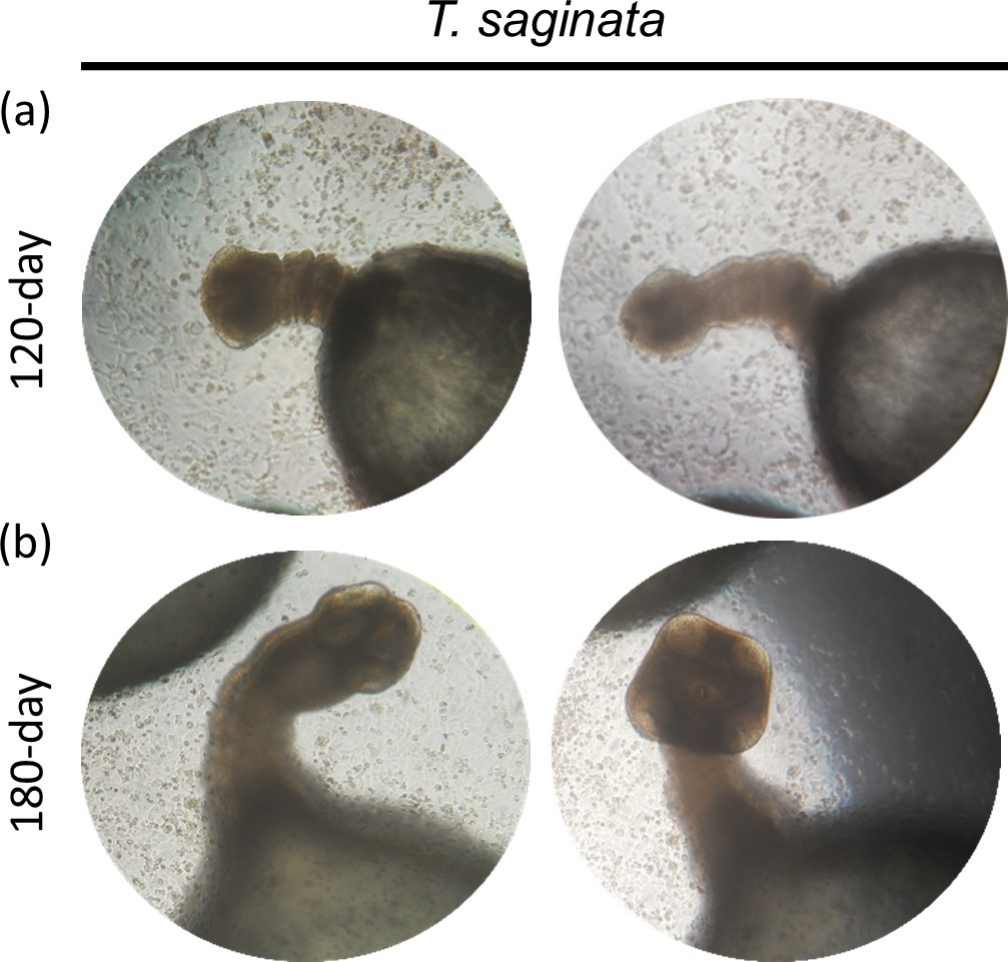
Light microphotography of *T*. *saginata* postoncospheral form at 120 and 180 days of culture in INT-407 cells. At 120 days of culture a pre-scolex-like structure appears (a) and at 180 days of culture, a scolex appears (b). Magnification 100x.

At day 120 of culture, *T*. *saginata* developed into a cysticercus with a spherical form and an evaginated pre-scolex containing four characteristic suckers and measuring 4 mm in diameter (Fig 2A). At day 180 of culture, *T*. *saginata* cysticerci increased in size compared to 120 day, reaching up to 6 mm and also displayed a well-defined scolex and suckers (Fig 2B).

### *T*. *solium* oncosphere and PO form resulted in NCC in rat infection

We infected rats with *T*. *saginata* oncosphere and PO forms in order to evaluate if *T*. *saginata* can develop into cysticercus *in vivo*, as *T*. *solium* does. Table 2 shows rats that were infected with *T*. *solium* oncosphere or 15-days PO form developed NCC, while rats that were infected with *T*. *saginata* oncosphere or 15-days PO form did not develop NCC.

**Table 2:** Percent of rats infected after intracranial inoculation with *T*. *solium* and *T*. *saginata* oncosphere and PO form.

Parasite form	% of rats infected with *T*. *solium*	% of rats infected with *T*. *saginata*
Oncospheres	67 (4/6)	0 (0/7)
15-day post oncospheres	63 (5/8)	0 (0/8)
Saline solution	0 (0/7)	0 (0/7)

### *T*. *solium* and *T*. *saginata* forms stimulate cytokines and MMP-9 in PBMC culture

Given that *T*. *saginata* does not develop to the cysticerci form in humans, we investigated the differences in host immune response to infection by *T*. *solium* and *T*. *saginata*. To determine this, we evaluated the production of cytokines during stimulation with AO and 30-day PO antigens of both species.

**Fig 3:**
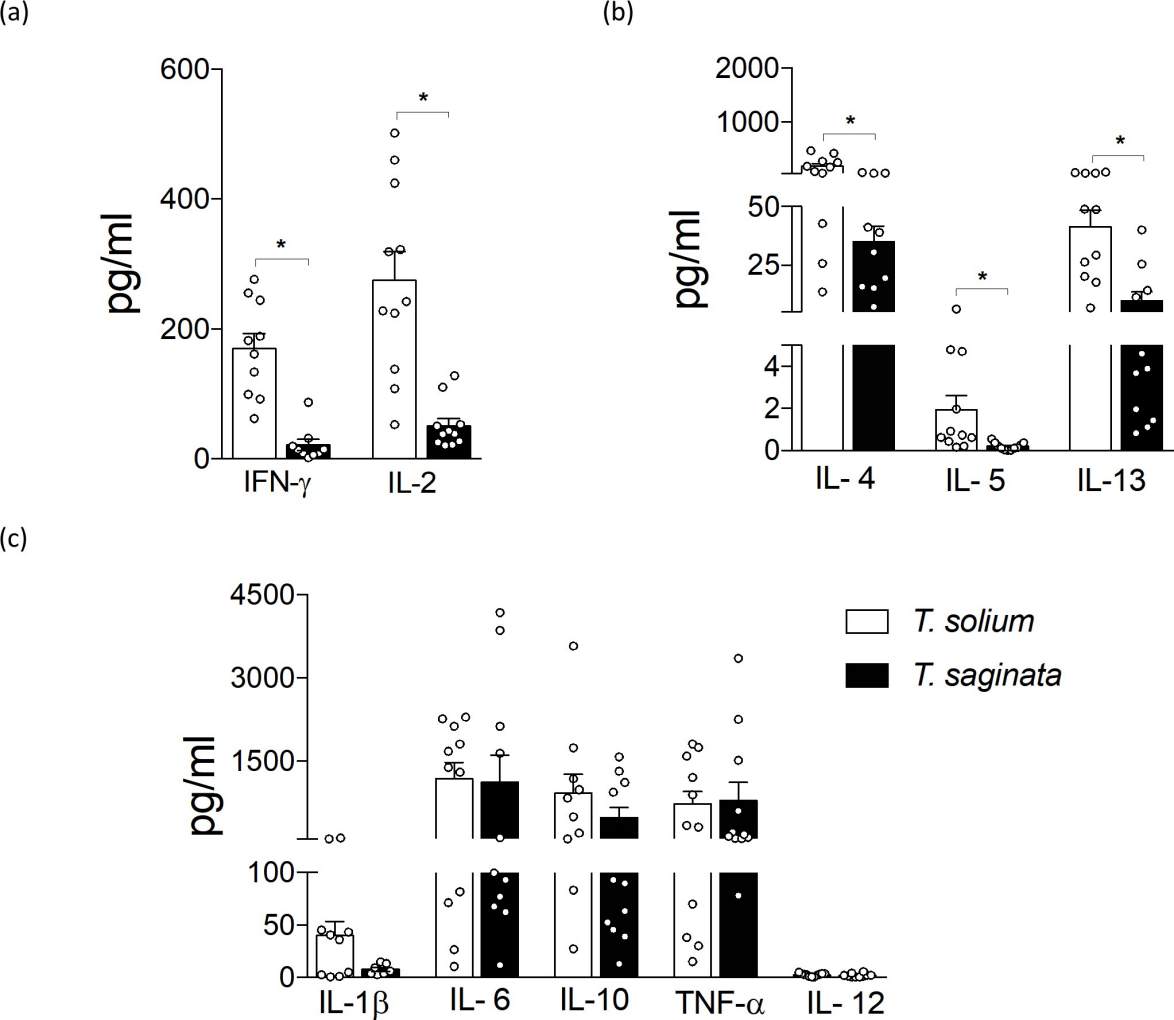
Production of cytokines by human PBMCs stimulated with *T*. *solium* and *T*. *saginata* AO form. Cultures supernatant were collected at 48 h from healthy human PBMC stimulated with 20 μg/ml of activated oncosphere antigens. (a) T_H_1- related cytokines, (b) T_H_2- related cytokines, and (c) other cytokines. Data presented are the mean ± SEM of healthy human donors (n = 10–13) minus control. * P< 0.05 was considered statistically significant.

**Fig 4:**
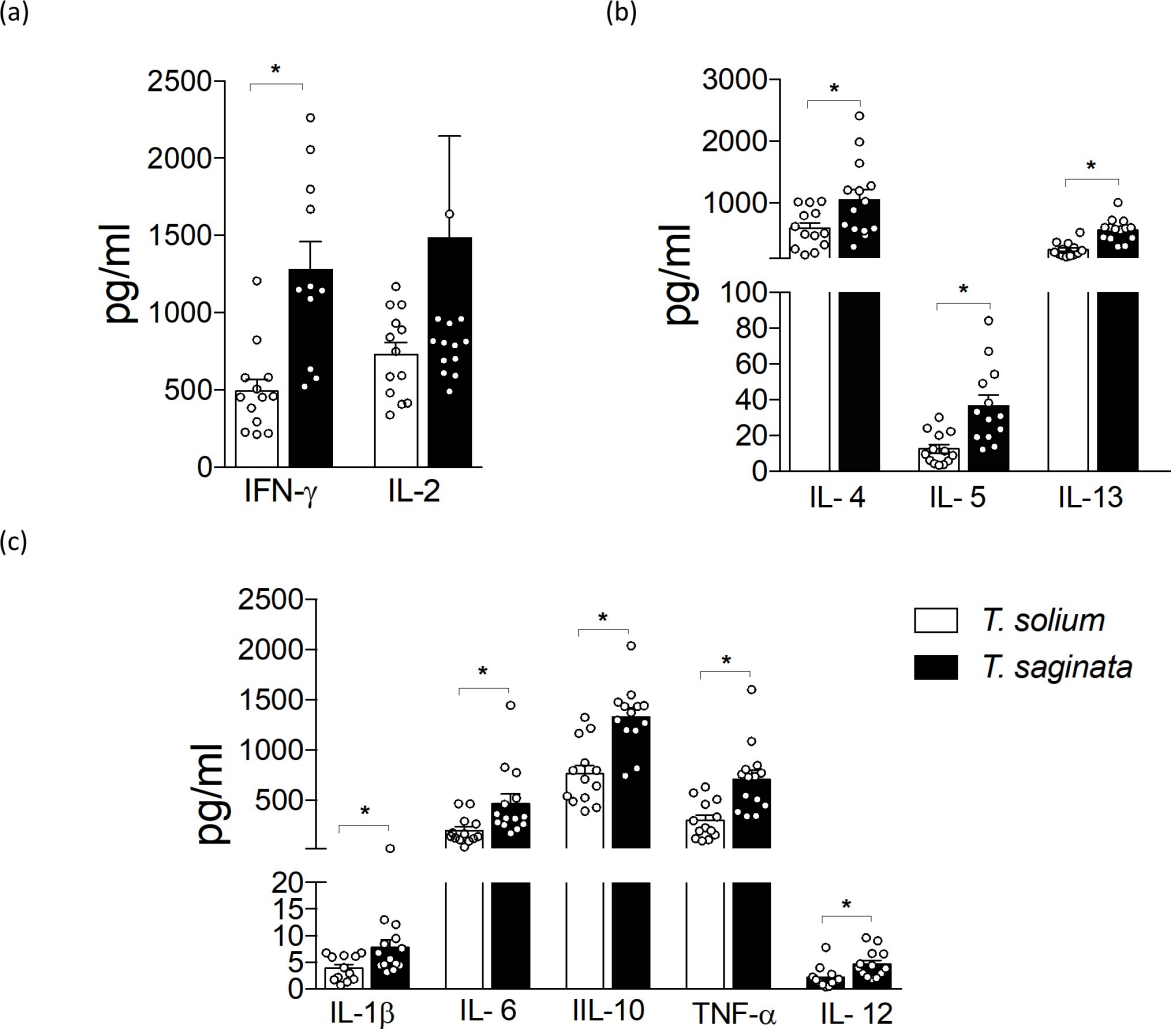
Production of cytokines by human PBMCs stimulated by *T*. *solium* and *T*. *saginata* PO 30-day forms. Cultures supernatant were collected at 48 h from healthy human PBMC stimulated with 20 μg/ml of 30-day PO form antigens. (a) T_H_1- related cytokines and (b) T_H_2- related cytokines, and (c) other cytokines. Data presented are the mean ± SEM of healthy human donors (n = 10–13) minus control. * P< 0.05 was considered statistically significant.

When compared by stages (AO and PO form) species, both *T*. *solium* and *T*. *saginata* PO forms stimulated a higher production of the majority of cytokines evaluated than the AO forms (S1 Fig). Nevertheless, when compared by species stages (AO and PO form), we observed a higher production of IL-4, IL-5, IL-13, IFN-γ and IL-2 cytokines stimulated by the *T*. *solium* AO form (Fig 3A and 3B) compared to the *T*. *saginata* AO; but in PO form, the *T*. *saginata* 30-day PO form stimulated a higher production of a variety of cytokines, including, IL-4, IL-5, IL-13, IFN-γ, IL-1β, IL-6, IL-10, TNF-α and IL-12 compared to the *T*. *solium* 30-day PO form (Fig 4A, 4B and 4C).

**Fig 5:**
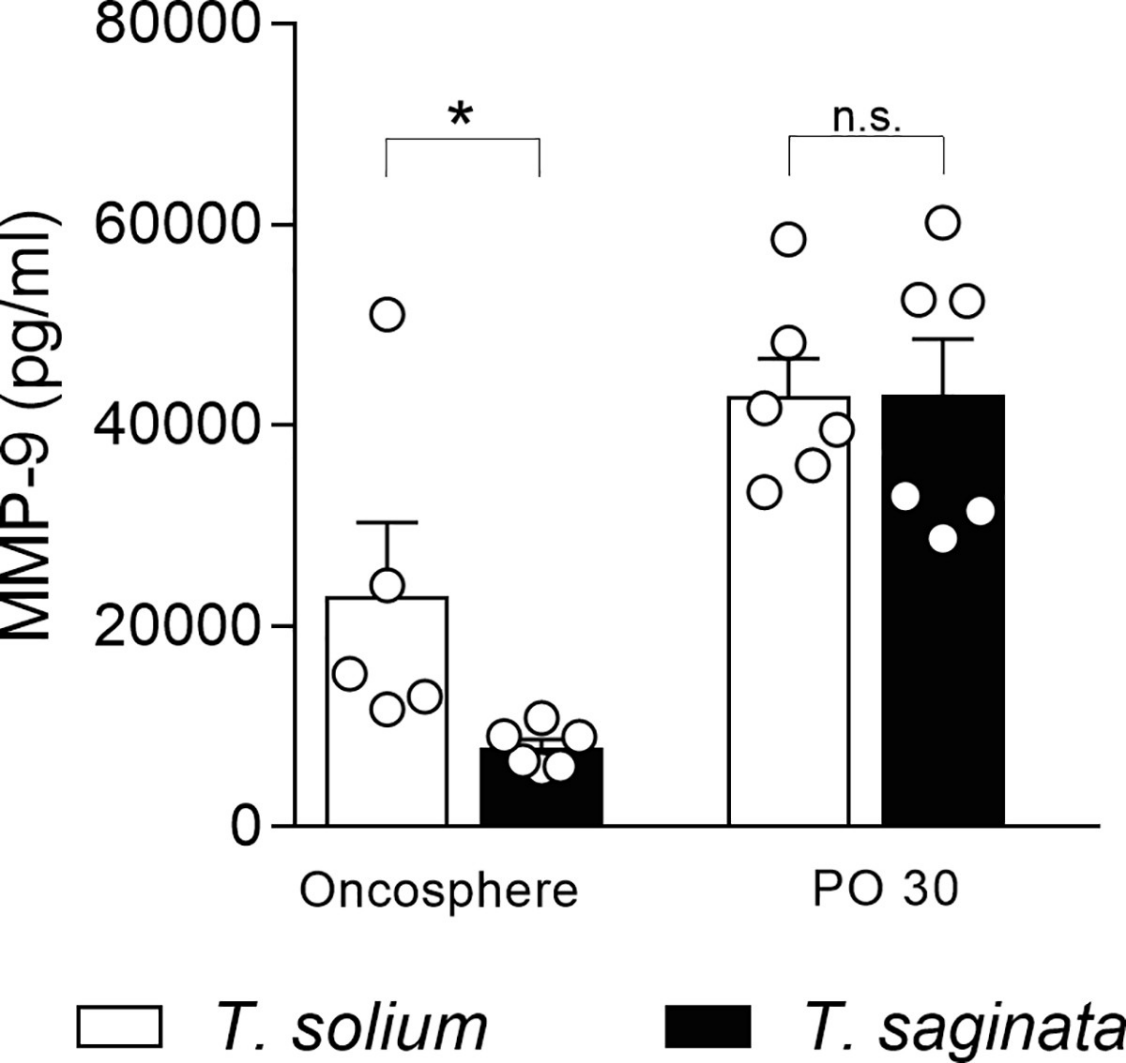
Production of MMP-9 by human PBMC stimulated with *T*. *solium* and *T*. *saginata* form. Culture supernatant was collected at 48 h from healthy human PBMC stimulated with 20 μg/ml of activated oncosphere antigens and 20 μg/ml of 30-day PO form antigens of *T*. *solium* and *T*. *saginata*. Data presented are the mean ± SEM of healthy human donors (n = 5–6) minus control. * P< 0.05 was considered statistically significant; n.s. = not significant.

To evaluate whether MMPs are involved in the host immune response against the parasites, we stimulated PBMCs with *T*. *solium* and *T*. *saginata* forms. The *T*. *solium* AO stimulated higher MMP-9 production compared to the *T*. *saginata* AO, while in PO form there was no significant difference between both species (Fig 5). There was no significant difference in the production of MMP-2 by PBMCs stimulated with different antigens of both parasites (S2 Fig).

## Discussion

The present study demonstrates that *T*. *saginata* activated oncospheres can develop into cysticerci *in vitro* in a human cell line, while *T*. *solium* AO do not. In contrast, in the rat model, the *T*. *saginata* oncosphere and PO forms did not develop to cysticerci *in vivo* as *T*. *solium* does. We suspect the differences in development between the two species in the *in vitro* model are due in large part to the environment of the growth media and the specific cell line, whereas *in vivo* it is the host immune response that plays a predominant role in regulating parasite development and survival. For instance, Heath and Smyth noted that serum used for *in vitro* culture contains factors unique to each host-parasite system, which can stimulate development of the parasite [16]. In our study, both species were cultured in media containing fetal bovine serum (FBS). FBS is known to contain large amounts of α- and β-globulin, proteins which have been shown to stimulate the development of the *T*. *saginata* oncosphere but not the development of other parasites like *T*. *taeniaformis* [17]. Additionally, the cell line used for culture likely plays an important role as *T*. *saginata* is able to develop to a cysticercus *in vitro* using cell line INT-407, while *T*. *solium* does not. On the other hand, *T*. *saginata* and *T*. *solium* do not develop into cysticercus using the HCT-8 cell line. INT-407 cells originate from the duodenum while the HCT-8 cell line originates from the colon. Furthermore, it is known that INT-407 cells are contaminated with HeLa cells that express different surface molecules that could promote the development of *T*. *saginata*. To our knowledge, this is the first study to achieve *in vitro* development of *T*. *saginata* PO forms to cysticerci.

In the *in vivo*, *T*. *solium* oncosphere and PO forms developed to cysticerci in brain while *T*. *saginata* forms did not. This finding is likely due to *T*. *solium’s* ability to evade the host immune response, either through binding of host plasma proteins or by synthesizing surface proteins that are antigenically similar to those of the rat [18]. Previous work has demonstrated that *T*. *taeniaeformis* oncospheres are able to initiate PO development after acquiring a component of rat serum on their surface, a component that presumably protects the oncosphere in the newly invaded host from recognition as a foreign antigen [17]. In the same study, *T*. *saginata* failed to bind this component and was subsequently attacked by the host immune system. A similar observation has been noted in pigs after infection with *T*. *saginata* eggs [19]. Another explanation may be genetic factors that play a role in the rat’s resistance to infection from *T*. *saginata* [20].

Although the rat is not a natural host of *T*. *solium*, we observed in previous studies that the cysticerci that develop in the rat brain are morphologically equal to those that develop in humans, as is the observed inflammatory response and subsequent pathology [11, 21]. Therefore, we believe the rat, like the human, has molecules that prevent the development of *T*. *saginata*.

The cytokines response plays an important role in survival of the oncosphere at the time of initial infection. We suspect *T*. *solium* stimulates a different cytokines profile than *T*. *saginata* allowing the *T*. *solium* oncosphere to survive, while *T*. *saginata* is destroyed by the host. To probe this hypothesis, we stimulated cytokine and MMP production in healthy human PBMCs using antigens of oncosphere and PO forms from both species.

We observed that *T*. *solium* oncospheres stimulate a higher production of IL-4, IL-5, IL-13, IFN- γ and IL-2 compared to *T*. *saginata* oncospheres. The IL-4, IL-5, IL-13, are cytokines typically associated with a T helper 2 (T_H_2) response, the predominant protective immune response against helminthic infections [22]. On the other hand, IFN- γ and IL-2 are associated with a T helper 1 (T_H_1) response, which is thought to be responsible for destruction of the parasite [23]. The T_H_2 response is important in protecting against extracellular helminthic parasites through suppression of the T_H_1 response, neutralization of toxins, and defense of the host against damage [22, 24].

Inflammatory reactions are dependent on a delicate balance between T_H_1 and T_H_2 type responses. In the case of the *T*. *solium* oncosphere, it appears a mix immune reaction of T_H_1/ T_H_2 type response that aids survival of the parasite. Perhaps in doing so, *T*. *solium* oncospheres are able to migrate from the vasculature to the brain, where they develop into the PO form. Similar mixed T_H_1/T_H_2 phenotypes have been observed in patients with NCC [7, 13] whereas a predominantly T_H_2-type response is associated with asymptomatic disease [25, 26].

In both species, the 30-day PO form generated an overall greater inflammatory response than the AO form. Levels cytokines increased when exposed to equal concentrations of PO vs AO antigen, suggesting that antigen composition changes as the parasite is maturing [9].

However, the *T*. *saginata* 30-day PO form stimulated a profile of pro-inflammatory cytokines (IL-1β, IL-6, IL-12, TNF-α), plus a mix of T_H_1 and T_H_2 related cytokines (IL-4, IL-5, IL-13 and IFN-γ) that was stronger than the response produced by the *T*. *solium* 30-day PO form. IL-6, a pro-inflammatory cytokine, plays a role in the death of microorganisms by stimulating the behavior of neutrophils [27]; and TNF-α is strongly expressed at the sites of parasite and cell destruction [28]. Together with the overproduction of IL-4, IL-5, and IL-13, these cytokines may mount a response that could destroy the *T*. *saginata* PO form and prevents development of the cyst *in vivo*. Although these cytokines do have the suggested properties mentioned, they also could be involved in complex networks with mixed effects with respect to inflammation, for example IL-6 has been shown to exhibit anti-inflammatory properties [29].

*T*. *solium* oncospheres stimulated increased MMP-9 production in PBMCs compared to production by *T*. *saginata* oncospheres. MMP-9 is an endopeptidase produced by neutrophils, macrophages, monocytes, and intestinal epithelial cells [30,31]. It can degrade components of the blood brain barrier (BBB) as well as the extracellular matrix, increasing intestinal epithelial permeability [30,32,33]. MMP-9 has been associated with the breakdown of the BBB in a murine model of NCC [34,35] and is present in high concentrations in sera of symptomatic NCC patients [36]. We hypothesize that *T*. *solium* stimulates the production of MMP-9 as a means of enhancing epithelial permeability in order to pass restrictive biological barriers like the intestine and the BBB during early stages of infection.

In conclusion, we have found novel evidence to suggest that *T*. *saginata* PO forms are capable of developing into cysticerci in the human cell line INT-407 while not in HCT-8 cells. *In vivo*, *T*. *saginata* fails to develop into cysticerci in the rat brain, suggesting there are factors in the host immune system (that are not present in the *in vitro* culture) that destroy the parasite. In the oncosphere stage, *T*. *solium* stimulated a strong mix of T_H_1 and T_H_2-related cytokines and MMP-9 production in healthy PBMCs, which may mediate the inflammatory response and promote oncosphere survival in the vasculature, aiding the entrance of oncospheres into the brain. In the PO form, *T*. *saginata* stimulated a strong pro-inflammatory and mix of T_H_1/T_H_2-related cytokines, responses that could be causing destruction of the parasite in the tissue. These differences between both species of Taenia found *in vitro* and *in vivo* could explain why the larval stage of *T*. *saginata* does not develop in the human host, while *T*. *solium* does, despite having similar life cycles.

## Supporting information

S1 Fig Production of cytokines by human PBMCs stimulated with AO and PO antigen. A) *Taenia solium* antigen B) *Taenia saginata* antigen.
(TIF)

S2 Fig Production of MMP-9 by human PBMC stimulated with *T*. *solium* and *T*. *saginata* form antigen. (TIF)

## References

[1] Okello AL, Thomas LF. Human taeniasis: current insights into prevention and management strategies in endemic countries. Risk Manag Healthc Policy. 2017;10:107–16. 10.2147/RMHP.S116545 28615981 PMC5461055

[2] Del Brutto OH. Neurocysticercosis: a review. ScientificWorldJournal. 2012;2012:159821 10.1100/2012/159821 22312322 PMC3261519

[3] Global Health—Division of Parasitic Diseases. CDC—Taeniasis—Enfermedad [Internet]. Centers Dis. Control Prev. 2013 [cited 2018 Sep 14];Available from: https://www.cdc.gov/parasites/taeniasis/es/enfermedad.html

[4] Garcia HH, Del Brutto OH. Neurocysticercosis: updated concepts about an old disease. Lancet Neurol. 2005;4(10):653–61. 10.1016/S1474-4422(05)70194-0 16168934

[5] Mahanty S, Garcia HH. Cysticercosis and neurocysticercosis as pathogens affecting the nervous system. Prog Neurobiol. 2010;91(2):172–84. 10.1016/j.pneurobio.2009.12.008 20035822

[6] Conn DB, Swiderski Z. A standardised terminology of the embryonic envelopes and associated developmental stages of tapeworms (Platyhelminthes: Cestoda). Folia Parasitol (Praha). 2008;55(1):42–52. 18578166 10.14411/fp.2008.006

[7] Tuero I, Palma S, Cabeza F, Saleemi S, Rodriguez S, Gonzales I, et al A Comparative Study of Peripheral Immune Responses to *Taenia solium* in Individuals with Parenchymal and Subarachnoid Neurocysticercosis. PLoS Negl Trop Dis. 2015;9(10):e0004143 10.1371/journal.pntd.0004143 26506532 PMC4624727

[8] Singh G, Prabhakar S. Taenia solium cysticercosis: from basic to clinical science Wallingford: CABI Pub; 2002

[9] Chile N, Clark T, Arana Y, Ortega YR, Palma S, Mejia A, et al In Vitro Study of *Taenia solium* Postoncospheral Form. PLoS Negl Trop Dis. 2016;10(2):e0004396 10.1371/journal.pntd.0004396 26863440 PMC4749246

[10] Schramlova J, Blazek K, Marhoul Z, Singhvi A. Ultrastructure of *Taenia saginata* oncospheres cultivated in artificial media. Folia Parasitol (Praha). 1984;31(3):247–51. 6479753

[11] Verastegui MR, Mejia A, Clark T, Gavidia CM, Mamani J, Ccopa F, et al Novel rat model for neurocysticercosis using *Taenia solium*. Am J Pathol. 2015;185(8):2259–68. 10.1016/j.ajpath.2015.04.015 26216286 PMC4530126

[12] Ll Terrazas. The complex role of pro- and anti-Inflammatory cytokines in cysticercosis: immunological lessons from experimental and natural hosts. Curr Top Med Chem. 2008;8(5):383–92. 18393901 10.2174/156802608783790848

[13] Amit P, Prasad KN, Kumar GR, Shweta T, Sanjeev J, Kumar PV, et al Immune response to different fractions of *Taenia solium* cyst fluid antigens in patients with neurocysticercosis. Exp Parasitol. 2011;127(3): 687–92. 10.1016/j.exppara.2010.11.006 21115005

[14] Jeri C, Gilman RH, Lescano AG, Mayta H, Ramirez ME, Gonzalez AE et al Species identification after treatment for human taeniasis. Lancet. 2004;363 (9413):949–50. 10.1016/S0140-6736(04)15791-7 15043964

[15] Verastegui M, Gilman RH, Arana Y, Barber D, Velasquez J, Farfan M, et al *Taenia solium* oncosphere adhesion to intestinal epithelial and Chinese hamster ovary cells in vitro. Infect Immun. 2007;75(11):5158–66. 10.1128/IAI.01175-06 17698575 PMC2168301

[16] Heath DD, Smyth JD. In vitro cultivation of *Echinococcus granulosus*, *Taenia hydatigena*, *T*. *ovis*, *T*. *pisiformis* and *T*. *serialis* from oncosphere to cystic larva. Parasitology. 1970;61(03):329–43. 4101140 10.1017/s0031182000041184

[17] Heath DD, Elsdon-Dew R. The in vitro culture of *Taenia saginata* and *Taenia taeniaeformis* larvae from the oncosphere, with observations on the role of serum for in vitro culture of larval cestodes. Int J Parasitol. 1972;2(1):119–30. 4119941 10.1016/0020-7519(72)90040-9

[18] Desowitz RS, Watson HJC. Studies on *Trypanosoma Vivax*. VI. The occurrence of antibodies in the sera of infected sheep and white rats, and their influence on the course of infection in white rats. Ann Trop Med Parasitol. 1953;47(3):247–57. 13105253

[19] Dorny P, Dermauw V, Van Hul A, Trevisan C, Gabriel S. Serological diagnosis of *Taenia solium* in pigs: No measurable circulating antigens and antibody response following exposure to *Taenia saginata* oncospheres. Vet Parasitol. 2017;245:39–41. 10.1016/j.vetpar.2017.08.008 28969835

[20] Fragoso G, Lamoyi E, Mellor A, Lomeli C, Govezensky T, Sciutto E. Genetic control of susceptibility to *Taenia crassiceps* cysticercosis. Parasitology. 1996;112(01):119–24. 8587794 10.1017/s003118200006515x

[21] Mejia Maza A, Carmen-Orozco RP, Carter ES, Dávila-Villacorta DG, Castillo G, Morales JD, et al Axonal swellings and spheroids: a new insight into the pathology of neurocysticercosis. Brain Pathol. 2018 10.1111/bpa.12669 30368965 PMC6482075

[22] Anthony RM, Rutitzky LI, Urban JF, Stadecker MJ, Gause WC. Protective immune mechanisms in helminth infection. Nat Rev Immunol. 2007;7(12):975–87. 10.1038/nri2199 18007680 PMC2258092

[23] Carpio A. Neurocysticercosis: an update. Lancet Infect Dis. 2002;2:751–62. 12467692 10.1016/s1473-3099(02)00454-1

[24] Mishra PK, Palma M, Bleich D, Loke P, Gause WC. Systemic impact of intestinal helminth infections. Mucosal Immunol. 2014;7(4):753–62. 10.1038/mi.2014.23 24736234

[25] Chavarria A, Fleury A, Bobes RJ, Morales J, Fragoso G, Sciutto E. A depressed peripheral cellular immune response is related to symptomatic neurocysticercosis. Microbes Infect. 2006;8(4):1082–9. 10.1016/j.micinf.2005.11.005 16520076

[26] Garcia HH, Nash TE, Del Brutto OH. Clinical symptoms, diagnosis, and treatment of neurocysticercosis. Lancet Neurol 2014;13(12):1202–15. 10.1016/S1474-4422(14)70094-8 25453460 PMC6108081

[27] Nadeau WJ, Pistole TG, McCormick BA. Polymorphonuclear leukocyte migration across model intestinal epithelia enhances Salmonella typhimurium killing via the epithelial derived cytokine, IL-6. Microbes Infect. 2002;4(14):1379–87. 12475628 10.1016/s1286-4579(02)00020-5

[28] Lima ES, Andrade ZA, Andrade SG. TNF-alpha is expressed at sites of parasite and tissue destruction in the spleen of mice acutely infected with Trypanosoma cruzi. Int J Exp Pathol. 2001;82(6):327–36. 10.1046/j.1365-2613.2001.00203.x 11846839 PMC2517787

[29] Scheller J, Chalaris A, Schmidt-Arras D, Rose-John S. The pro- and anti-inflammatory properties of the cytokine interleukin-6. Biochimica et Biophysica Acta (BBA)—Molecular Cell Research. 2011;1813(5):878–88. 21296109 10.1016/j.bbamcr.2011.01.034

[30] Bruschi F, Pinto B. The Significance of matrix metalloproteinases in parasitic infections involving the central nervous system. Pathogens. 2013;2(1):105–29. 10.3390/pathogens2010105 25436884 PMC4235708

[31] Gan X, Wong B, Wright SD, Cai TQ. Production of matrix metalloproteinase-9 in CaCO-2 cells in response to inflammatory stimuli. J Interferon Cytokine Res. 2001;21(2):93–8. 10.1089/107999001750069953 11244573

[32] Parks WC, Wilson CL, Lopez-Boado YS. Matrix metalloproteinases as modulators of inflammation and innate immunity. Nat Rev Immunol 2004;4(8):617–29. 10.1038/nri1418 15286728

[33] Nighot P, Al-Sadi R, Rawat M, Guo S, Watterson DM, Ma T. Matrix metalloproteinase 9-induced increase in intestinal epithelial tight junction permeability contributes to the severity of experimental DSS colitis. Am J Physiol Liver Physiol. 2015;309(12):988–97. 10.1152/ajpgi.00256.2015 PMC4683300 26514773

[34] Alvarez JI, Teale JM. Multiple expression of matrix metalloproteinases in murine neurocysticercosis: Implications for leukocyte migration through multiple central nervous system barriers. Brain Res. 2008;1214:145–58. 10.1016/j.brainres.2008.03.036 18466882 PMC2517245

[35] Alvarez JI, Teale JM. Differential changes in junctional complex proteins suggest the ependymal lining as the main source of leukocyte infiltration into ventricles in murine neurocysticercosis. J Neuroimmunol. 2007;187(1–2):102–13. 10.1016/j.jneuroim.2007.05.005 17597230 PMC2692657

[36] Verma A, Prasad KN, Nyati KK, Singh SK, Singh AK, Paliwal VK, et al Association of MMP-2 and MMP-9 with clinical outcome of neurocysticercosis. Parasitology. 2011;138 (11):1423–8. 10.1017/S0031182011001259 21813044

