# Supplementary figures and images for: In vitro model of postoncosphere development, and in vivo infection abilities of Taenia solium and Taenia saginata
Source: PLoS Negl Trop Dis. 2019 Mar 14;13(3):e0007261. doi: 10.1371/journal.pntd.0007261 (PMC6435196; doi:10.1371/journal.pntd.0007261)

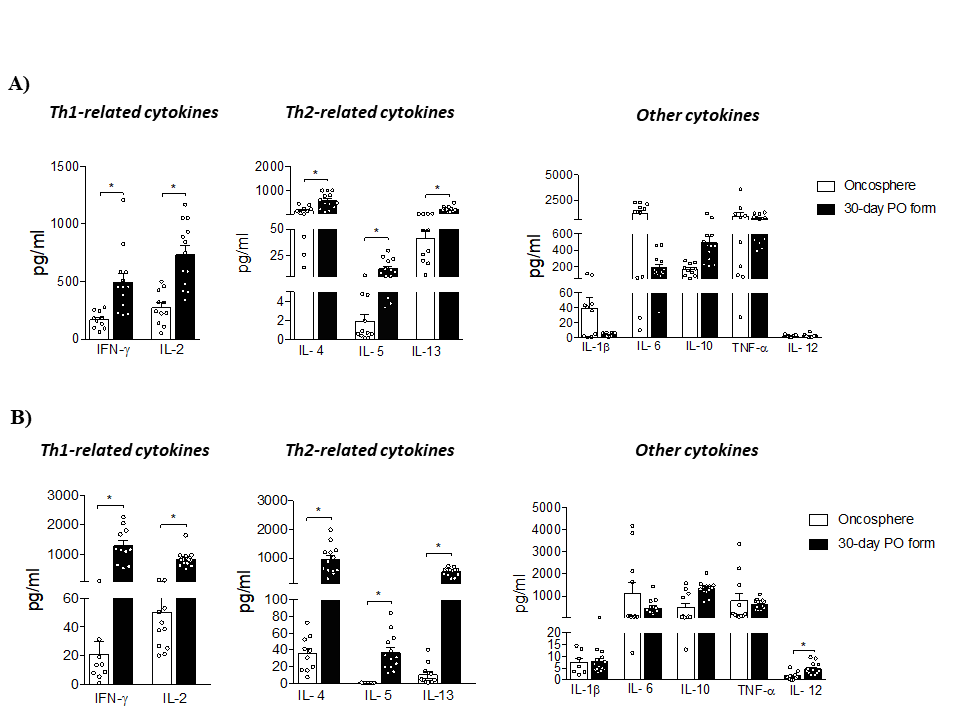

Supplement: S1 Fig — A) Taenia solium antigen B) Taenia saginata antigen. (TIF) [file pntd.0007261.s001.tif]

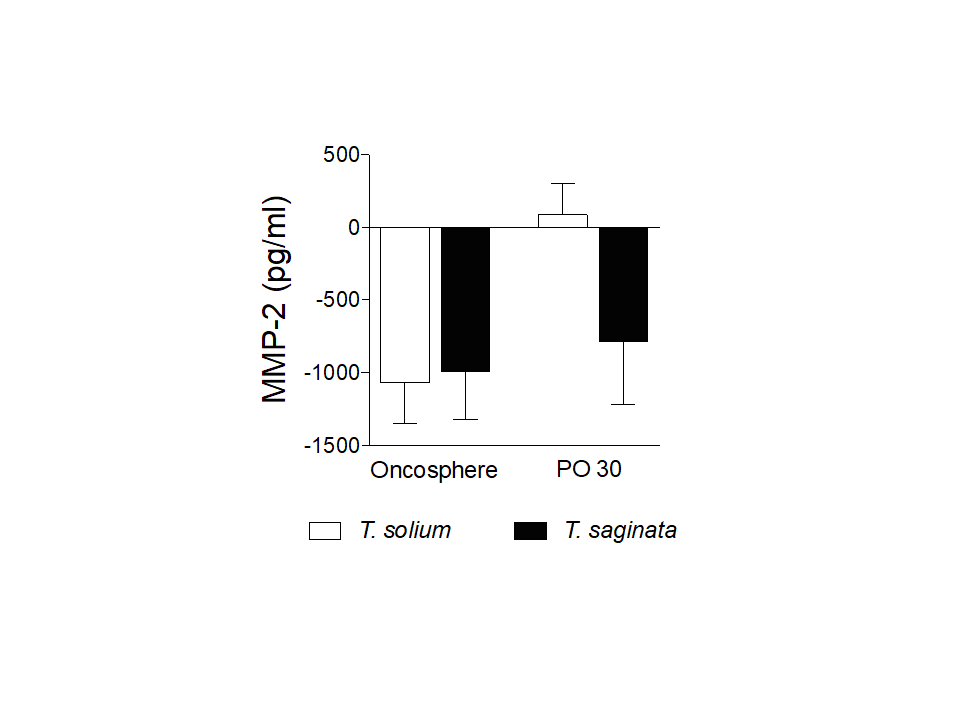

Supplement: S2 Fig — (TIF) [file pntd.0007261.s002.tif]
